# Development and validation of an adolescent health literacy scale in Ethiopia: A mixed methods approach

**DOI:** 10.1371/journal.pone.0329184

**Published:** 2025-08-08

**Authors:** Adamu Amanu Asari, Ameyu Godesso, Zewdie Birhanu

**Affiliations:** 1 Department of Health, Behavior, and Society, Faculty of Public Health, Jimma University, Oromia, Ethiopia; 2 Department of Sociology, College of Social Sciences, Jimma University, Oromia, Ethiopia; Jimma University, ETHIOPIA

## Abstract

**Background:**

Adolescent health literacy (HL) is a significant public health concern, especially in developing countries with predominantly young populations like Ethiopia, as it impacts both their immediate and long-term health outcomes, and consequently, the overall wellbeing of their societies. Since HL is influenced by socio-cultural contexts, a locally developed and validated HL scale is essential for generating quality data and designing effective interventions. However, such a context-specific, validated HL scale for this age group is currently lacking in Ethiopia. This study, therefore, aims to develop and validate an adolescent HL scale tailored to the socio-cultural context of Ethiopia, to support measurement and intervention efforts.

**Methods:**

This study employed a mixed methods approach, conducted in three stages between March 09, 2023 and January 25, 2024. In the first stage, concepts, categories/dimensions, and items were generated, using in-depth interviews and focus group discussions as data collection methods, to inform scale design, and the scale was subsequently developed based on these elements. The second stage comprised evaluating the scale’s content, cognitive, and usability standards through expert reviews/evaluations, cognitive interviews, and a pilot survey, followed by modifications based on these evaluations. The third stage involved further evaluation of the validity and reliability of the scale on a larger random sample, using confirmatory factor analysis and reliability tests, respectively. Purposive, convenience, and random sampling techniques were employed to select participants. The study was conducted in Afaan Oromo and Amharic languages as per the participants’ preferences. Data analysis was conducted using Atlas.ti (version 7.5.18) and SPSS with AMOS (version 23.0).

**Results:**

In the first stage of the study, an initial pool of 88 items was generated and then reduced to 40 items following a rigorous review of their coverage and uniqueness. In the second stage, evaluations for content, cognitive, and usability standards further refined the items to 33 covering five dimensions: health information competency, communication, health awareness and knowledge, decision-making and behavior, and citizenship and responsibility. Finally, in the third stage, validity and reliability testing of the 33-item scale yielded strong results. For instance, confirmatory factor analysis produced CFI, TLI, and RMSEA values of 0.976, 0.974, and 0.034, respectively, and reliability testing provided a Cronbach’s alpha of 0.973 for the full scale and 0.850–0.937 for the subscales in the Afaan Oromo version. For the Amharic version, the corresponding values were 0.965, 0.962, and 0.039, with Cronbach’s alpha values of 0.970 for the full scale and 0.783–0.937 for the subscales.

**Conclusion:**

The study developed and validated an adolescent HL scale, using a mixed methods approach within the socio-cultural context of Ethiopia, addressing a critical gap in this area. This scale is multidimensional, encompassing HL knowledge, skills, abilities, and related attributes or qualities essential for adolescents today. The scale can be adapted and used with populations beyond adolescents and in other contexts in future studies. It is also vital for designing effective strategies aimed at improving HL in adolescents.

## Background

HL is a key public health issue and a multifaceted concept [1–4]. It comprises the knowledge and abilities needed to interact effectively with the healthcare system as well as understanding the factors that influence health, knowing how to manage or change them, and the ability to make sound health decisions in the context of everyday life [2–6]. HL enables people to manage, protect, and enhance their health [2,7]. It is an empowering resource that allows individuals to make informed health decisions in their daily lives [2,5,8]. Hence, individuals having high HL status have better health status [9–12], while those with low HL status have poor physical and mental health and limited life opportunities [13–15]. Among adolescents, studies from various countries indicate that low HL is associated with risky health behaviors such as substance abuse, unsafe sexual practices, unhealthy diets, and other related actions [16–18]. This is concerning, as risky behaviors adopted during this period of life often have lifelong consequences [19–22].

Thus, adolescent HL is a critical public health concern [8,16], especially in developing countries with predominantly young populations like Ethiopia [23]. It impacts not only adolescents’ immediate and long-term health outcomes but also the overall wellbeing of their societies [8,24–26]. Adolescents need to be empowered with HL to adopt healthy behaviors, engage effectively with their health, and invest in the well-being of their communities [8,26].

As awareness of this issue increases, research on adolescent HL has been expanding [8,16,27]. However, there have been challenges in establishing universally acceptable definition, framework, and measurement of HL in adolescents [8,28–30]. In a systematic review of definitions and models of HL in childhood and youth, Bröder et al. [8] described HL in children and young people as comprising clusters of related abilities, skills, commitments, and knowledge that enable a person to approach health information competently and effectively and to derive at health-promoting decisions and actions. They also noted HL as encompassing various dimensions and attributes, including cognitive attributes (such as knowledge, basic or functional health-related skills, comprehension and understanding of health information, and appraisal and evaluation of health information), behavioral or operational attributes (such as seeking and accessing health information, communication and interaction, application of health information, and other context-specific skills for the application of health information, including citizenship), and affective and conative attributes (such as self-awareness, self-efficacy, and other related qualities) [8]. Their work has been instrumental in shaping the understanding of HL in young populations, laying a foundation for further research, including this study, and informing interventions to improve HL in adolescents.

To support measurements and interventions tailored to this age group, a number of HL tools also have been developed and validated over the past two decades [28,29,31–36]. However, these tools have been criticized for various reasons, including for not being comprehensive [31,32], for being general and very broad [35,37], for being adapted from that of adults and hence are inappropriate for adolescents’ context [31,32], for lacking quality [28,38], and for not being grounded in qualitative data or adolescents’ perspectives [28,31,32,35,38]. More recently, several HL tools have been introduced addressing a number of these limitations [39–41]. However, generally, HL tools have been developed and validated within the context of developed countries [28,36,39–41]. In developing countries like Ethiopia, there remains a lack of locally developed and validated HL tools, both for adolescents and the general population [42–45].

Since HL is specific to socio-cultural contexts [24,30], quality and meaningful data for effective interventions could only be obtained if HL measures reflect local sociocultural contexts [42]. In addition to individual attributes, HL is shaped by culture and society, the education system, and the health system [24,46]. Therefore, tools developed in high-income countries may not be appropriate for use in developing countries, due to differences in health beliefs, lifestyles, norms, values, as well the education, economic, and healthcare systems [42]. Developing and validating a HL scale in developing countries like Ethiopia is crucial for capturing context-specific perspectives, advancing the field of HL, informing targeted interventions, and guiding measurement practices. Therefore, this study aims to develop and validate an adolescent HL scale (AHLS) that is grounded in qualitative data from adolescents and tailored to the socio-cultural context of Ethiopia, with the goal of supporting interventions and measurements.

## Methods

### Study setting and population

The study took place in Jimma city, involving adolescents in schools. Jimma city is one of the oldest and largest cities in Oromia, Ethiopia, and it is known for its socioeconomically diverse population. The languages that are widely spoken in the area are Afaan Oromo and Amharic. The study area included 16 high schools during the period of the study. Adolescents from 11 high schools participated in this study at different stages between March 09, 2023 and January 25, 2024. The participating schools enrolled adolescents from diverse sociodemographic backgrounds, comprising those who were from urban and rural origins. At the time of the study, 12,542 students (5630 males and 6912 females) were enrolled in these schools.

### Study approach and stages

This study used a mixed methods approach to attain its purpose [47,48]. It employed a grounded theory method to generate concepts, categories, and items for the scale design from the adolescents’ perspectives [47,49,50]. Subsequently, the scale was designed/developed based on these generated concepts, categories, and items, and then evaluated and validated both qualitatively and quantitatively through the participation of experts and adolescents [47,50,51]. These were all accomplished in three stages. The following sections describe each stage separately, focusing on the methods used and the procedures followed, while the results are presented in the ‘Results’ section.

#### Stage I. Concepts, categories, and items generation, and scale design/development.

At this stage, the study employed in-depth interviews and focus group discussions (FGDs), guided by a grounded theory, to explore the adolescents’ HL perspectives and practices [26] and to generate concepts, categories, and items for the scale design. Purposive sampling with a maximal variation sampling strategy was used to select participants, ensuring diversity among participants in terms of sex, age, grade/class level, parental socioeconomic factors, and other relevant characteristics [26,47].

As described in [26], at this stage, the study used a data collection guide, which was constructed based on the aim of the study and insight gained from HL literature, and then tested for clarity and appropriateness. During the interviews and FGDs, subsequently, the adolescents were asked about HL and what it means to be health literate (including its characteristics and qualities), their practices regarding the issue, and related matters to explore and generate HL related concepts, categories/dimensions, and items for the intended purpose. The interviews and FGDs were carried out face-to-face in comfortable places within school compounds (either in free class rooms or outside the class rooms) in either Afaan Oromo or Amharic, based on the participants’ language preferences. The responses of the participants were rephrased, summarized, and reflected back to them to avoid misunderstandings and misinterpretations.

Sampling and data collection continued until data saturation was reached, that is, no further new information and concept emerged from participants [26,52,53]. In total, 86 male and female adolescents, aged 14–19 years old participated in the study at this stage. Forty seven of them participated in in-depth interviews (22 males & 25 females), and the remaining 39 participated in six separate FGDs (19 males & 20 females). Almost all of the interviews and FGDs were audio recorded based on the participants’ permissions, and the duration of the conversations varied from about 24–75 minutes. Fieldnotes were also recorded during the data collection process to enrich the study [26].

After completion, each interview and FGD was translated word by word. Then, the translated text was sorted and segmented; important quotes and segments were identified; the segments were coded; codes were categorized, and categories were grouped into broader categories/dimensions [49,52,54] using Atlas.ti version 7.5.18 software. This informed the generation and development of HL concepts, categories, and items for the scale design/development (See the ‘Results’ section). The researcher approached the data with an open mind over the course of the study [49], while acknowledging the influence of key concepts form HL literature, particularly [2,8] (See S1 File).

Following the generation and then revision of the concepts, categories/dimensions, and items according to recommended procedures [47,51], the first draft of the AHLS was designed, as detailed in the ‘Results’ section. The scale was primarily designed with a four-point Likert format (strongly disagree, disagree, agree, and strongly agree), with alternative Likert formats also considered as potential options. The scale was designed in both Afaan Oromo and Amharic languages.

#### Stage II. The scale’s content, cognitive, and usability standards evaluation.

Following its design, the scale needs to be evaluated on the basis of its content, cognitive, and usability standards ─ the standards all survey questions should meet [55]. This is to test whether, for instance, the questions asked about the right things (content standards), whether respondents understand the questions properly, are able and willing to answer them (cognitive standards), and whether they can complete the questionnaire easily and as intended (usability standards) [55]. For this purpose, this study employed expert reviews and evaluations, cognitive interviews, and pilot survey techniques, as recommended by Groves et al. [55], with the participation of totally 18 experts and 101 adolescents (43 males & 58 females, aged 14–19) as described below.

Experts from various academic fields, including public health, education, communication, and languages, were invited to evaluate the scale. Seven experts qualitatively evaluated the scale’s standard, particularly regarding the wording of the questions and the coverage and response alternatives. Eleven experts quantitatively evaluated the scale’s content validity, determining the necessity of each item on a 3-point Likert scale (1 = not essential, 2 = useful but not essential, 3 = essential) and its relevance on a 4-point Likert scale (1 = not relevant, 2 = somewhat relevant, 3 = relevant, and 4 = very relevant). The content validity ratio (CVR) and Content Validity Index (CVI) respectively were then calculated. As per Lawshe’s table [56] and K Hyrkäs et al. [57], items with CVR score ≥ 0.59 and items with CVI score > 0.79 respectively were considered as acceptable items.

Cognitive interviews were conducted with 20 adolescents (9 males & 11 females) selected using purposive sampling to evaluate the scale. The participants completed the drafted questionnaire and were interviewed (in Afaan Oromo or Amharic based on their preferences) to evaluate their thought process or what they think while reading the questions, to assess the clarity of the questions, including their views regarding the response options (Likert-scale format) used, to identify any problems/discomfort experienced, and to make required modifications accordingly.

Then, for the pilot survey, questionnaires were distributed to 82 adolescents selected using convenience sampling, and 81 questionnaires were filled out: 42 Afaan Oromo and 39 Amharic versions (by 34 male & 47 female respondents). The pilot survey evaluated several aspects of the scale, including its format, the order of the questions, and whether answers to the questions produce valid measurements. The pilot survey data were entered into database using Epidata 3.1 and analyzed using SPSS software version 23.0. The entered data were tabulated for each item to identify items with high rates of missing values, which could indicate problematic questions, particularly if more than 5% of values are missing [39]. Ceiling effect and floor effect were also determined for each item by calculating the percentage frequency scores of the highest and lowest values scored, as this suggests the scale might be too easy (not challenging enough) and too difficult respectively for the target population. A potential ceiling or floor effect was noted present when more than 15% of respondents scored at the extreme ends [58]. Reliability coefficient was also calculated using Cronbach’s alpha to ensure that the items have good internal consistency, and a Cronbach’s alpha coefficient of ≥ 0.70 was considered as indicative of acceptable reliability [59–61].

#### Stage III. Validity and reliability reassurance.

After successfully completing the aforesaid processes and making the required improvements, a cross-sectional survey was conducted to further evaluate the (construct) validity and internal reliability of the scale.

Based on a minimum sample size (n) recommended for this kind of study (n > 200) [62–66], a total of 620 adolescents were recruited randomly and invited to complete a self-administered questionnaire. And 611 completed questionnaires (by 277 male & 334 female respondents) were recollected (350 Afaan Oromo and 261 Amharic) (as one was missed and eight were incomplete).

Then, after cleaning the data, confirmatory factor analysis (CFA) was conducted using SPSS with AMOS software version 23.0 to evaluate the (construct) validity of the scale, as item dimensions or categories had already been identified/specified [67] in the first stage of this study. The following statistical measures were considered: chi-square test of model fit (χ2/df), Comparative Fit Index (CFI), Incremental Fit Index (IFI), Tucker-Lewis Index (TLI), Root Mean Square Error of Approximation (RMSEA), and Standardized Root Mean Square Residual (SRMR) [68–72]. For these measures (in the order listed), thresholds of < 5, > 0.90, > 0.90, > 0.90, < 0.08, and < 0.08 are indicators of acceptable fit, and values of < 3, > 0.95, > 0.95, > 0.95, < 0.06, and < 0.05 are indicators of excellent fit [71,73–76].

Reliability of the scale was evaluated by conducting internal consistency and test-retest reliability (stability) analyses. To determine the internal consistency of the scale, the Cronbach’s alpha coefficient was calculated for the whole scale and its subscales. A Cronbach’s alpha coefficient of 0.70 or above was noted as indicating acceptable reliability [59,77–79]. To assess the scale’s time stability, test-retest was conducted, with 64 additional adolescents (30 males & 34 females). These participants completed the questionnaire twice, two weeks apart [80,81]. And Intra-class Correlation Coefficients (ICC) (95% confidence interval) were calculated for both the entire scale and its subscales, with the understanding that an ICC value of ≥ 0.4 is acceptable, and an ICC value of ≥ 0.61 indicates good reliability [58,80,82].

### Ethical issues

The study was authorized by the ethics committee (Institutional Review Board) of Jimma University Institute of Health (Ref. No. JUIH/IRB/321/23). A letter of support regarding the issue was also obtained from the Department of Health, Behavior, and Society, and permission was secured from each school director. The researcher (the first author) explained the study’s aim to the target adolescents and obtained informed consent/assent from the participants. Participants aged ≥ 18 years provided direct informed consent, while those aged < 18 years, gave their assent after informed consent was secured from their parents or with whom they live, with school directors facilitating the process. The participants engaged in the study within their respective school compounds at convenient times and in comfortable settings. All participants were informed that they were under no obligation to participate and could withdraw from the study at any stage. They were also assured that their information would remain confidential in all reports and publications.

## Results

### Sociodemographic characteristics

In total, 862 adolescents participated in this study from stage one to stage three. There were 391 (45.36%) male and 471 (54.64%) female participants. The mean age of participants was 16.75 ± 1.28 years (between 14 and 19). Of the total participants, 626 (72.62%) were from public schools, and 236 (27.38%) were from private schools. In terms of origin, 612 (71.00%) and 250 (29.00%) of the participants had urban and rural backgrounds, respectively. Regarding religion, 415 (48.14%) were from Islam, 247 (28.65%) were from Orthodox, 178 (20.65%) were from Protestant, and 22 (2.55%) were from ‘Others’ (including Catholic and Seventh Day Adventist). Overall, the participants’ parents’ education status varied from unable to read and write to degree/above. They reported their fathers’ education as unable to read and write 107 (12.41%), primary school 224 (25.99%), secondary school 180 (20.88%), diploma 62 (7.19%), and degree/above 143 (16.59%), and mothers’ education as unable to read and write 145 (16.82%), primary school 264 (30.63%), secondary school 170 (19.72%), diploma 71 (8.24%), and degree/above 98 (11.36%). The remaining participants, 146 (16.94%) and 114 (13.23%) reported they ‘don’t know their fathers’ and mothers’ educational statuses, respectively. Parents’ occupation statuses varied accordingly. They reported that their fathers’ occupations were merchant 252 (29.23%), government employee 219 (25.41%), farmer 258 (29.93%), and others 133 (15.43%) (Taxi driver, carpenter, NGO worker, religious leader, and retired), and mothers’ occupations were merchant 209 (24.25%), government employee 155 (17.98%), farmer 96 (11.14%), housewife 360 (41.76%), and others 42 (4.87%) (including NGO worker and sanitation worker) (See S2 File)

### Concepts, categories, and items generation, and scale design/development

The results of in-depth interviews and FGDs in the first stage of the study revealed diverse HL perspectives among adolescents, reflecting the state of their HL knowledge, skills, abilities, and related attributes [26]. The core HL elements explored here include health awareness and knowledge, health information-related competency, communication, decision making, health-related behavior, and a sense and practice of citizenship and responsibility. These findings led to the development of five HL dimensions, namely, health information competency (ability to effectively access, understand, evaluate, and use health information), communication (ability to communicate or discuss health concerns with family, healthcare providers, and others), health awareness and knowledge (regarding health-related behaviors and consequences, health risk factors, and diseases, prevention, and treatment, including valuing health information), decision-making and behavior (ability to use health information to make informed decisions and adopt and practice healthy behaviors), and citizenship and responsibility (recognizing that one’s health behaviors and decisions affect others and acting accordingly, demonstrating and promoting healthy behaviors, and actively engaging in activities that support community health and well-being). These concepts and dimensions informed the development of an initial 88-item pool for the scale design. After rigorously reviewing whether the generated items are distinct from each other, deal with their respective dimensions, and cover HL knowledge, skills, abilities, and related qualities essential for adolescents, they were reduced to 40 items, forming the first draft of the AHLS, which was then ready for evaluation (See S3 File).

The following table (Table 1) illustrates how quotes from in-depth interviews and FGDs informed the development of HL concepts, dimensions, and items in this study.

**Table 1:** Development of HL concepts, dimensions, and items based on insights from interviews & FGDs.

Some representative quotes	Some important codes (concepts)	Dimensions/categories	Some items (and their standardized factor loading values)
*“….I search and learn health issues from Google.”* (Female, 16 years old) *“I often get health information from my uncle* [a health professional]*. He openly discusses with me...”* (Female, 17 years old) *“…I can understand what they tell me, and I ask for clarification if it is not clear.”* (Female, 17 years old) *“I search and understand it from Internet…. not only about health, about life in general.”* (Female, 16 years old) *“…It is better to confirm the validity of the information obtained before accepting and sharing it.”* (Male, 17 years old) *“…Valid information can be identified using Internet…”* (Male, 19 years old) *“…I don’t think distancing myself from something that harms me is difficult…it is good to have patience and avoid making decisions you might regret later.*” (Female, 18 years old) “*… I do not have sufficient information about HIV/AIDS, adolescence…*” (Female, 14 years old) *“…It is very important to be aware and protect oneself ….. as our school, sexually transmitted diseases issue is not a serious matter, as information about it is becoming repetitive; we need information about mental health, chronic diseases…”* (Male, 19 years old) *“…I think that those who engage in risky behaviors are not aware of the consequences in their later life.”* (Male, 15 years old) *“Many adolescents engaging in risk behaviors have knowledge about the problem, but they ignore it for the sake of temporary pleasure (fataa jechuumaafi).”* (Female, 16 years old) *“I don’t often find myself in conflict with others, this good behavior, but I often could not openly express what is in me…., this bad.”* (Female, 19 years old) “*Health information is needed for me…..I also want to help others; there are those who are in different problems and are unable to live for their purposes. I want to help such like individuals.* (Female, 16 years old) *“…. We have to learn it for ourselves, our family, and our country.”* (Female, 16 years old)	▪ Accessing ▪ Understanding ▪ Appraising ▪ Applying ▪ Communication ▪ Awareness ▪ Knowledge ▪ Decision-making ▪ Behavior ▪ Responsibility ▪ ‘Citizenship’	▪ Health information competency (HIC) ▪ Communication (COM) ▪ Health awareness and knowledge (HAK) ▪ Decision-making and behavior (DMB) ▪ Citizenship and responsibility (CR)	HIC2-You are able to access health information you need from various sources. (0.665, See Fig 1 and S4 and S5 Files) HIC6-You can easily understand the health information you obtain from various sources. (0.642, See Fig 2 and S4 and S6 Files) COM3-You can openly discuss any health concerns you have, including issues related to adolescence and RH, with others whom you believe have knowledge of or experience in the matter. (0.820, See Fig 1 and S4 and S5 Files) HAK2-You have adequate information for your age regarding healthy and unhealthy behaviors. (0.729, See Fig 2 and S4 and S6 Files) DMB3-You take care of or prioritize your health every day, based on information you have obtained, regardless of the conditions. (0.748, See Fig 1 and S4 and S5 Files) CR2-You share your health knowledge with friends and help them avoid risky behaviors, such as addiction, and adopt healthy habits. (0.758, See Fig 2 and S4 and S6 Files)

**Fig 1:**
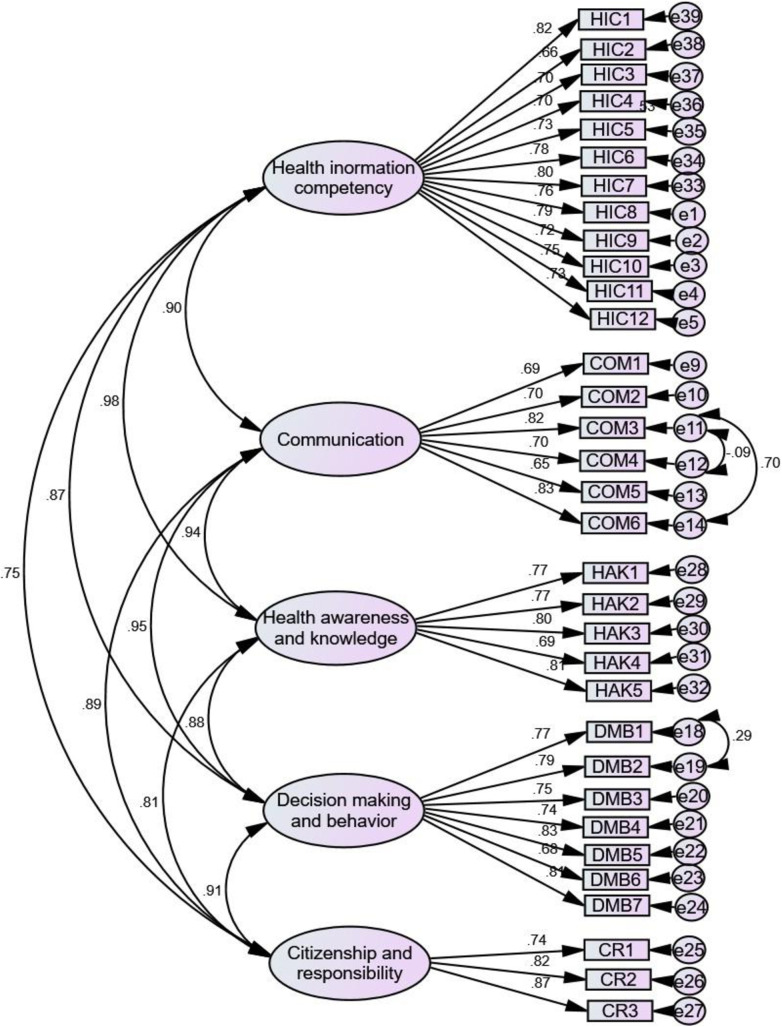
Afaan Oromo version.

**Fig 2:**
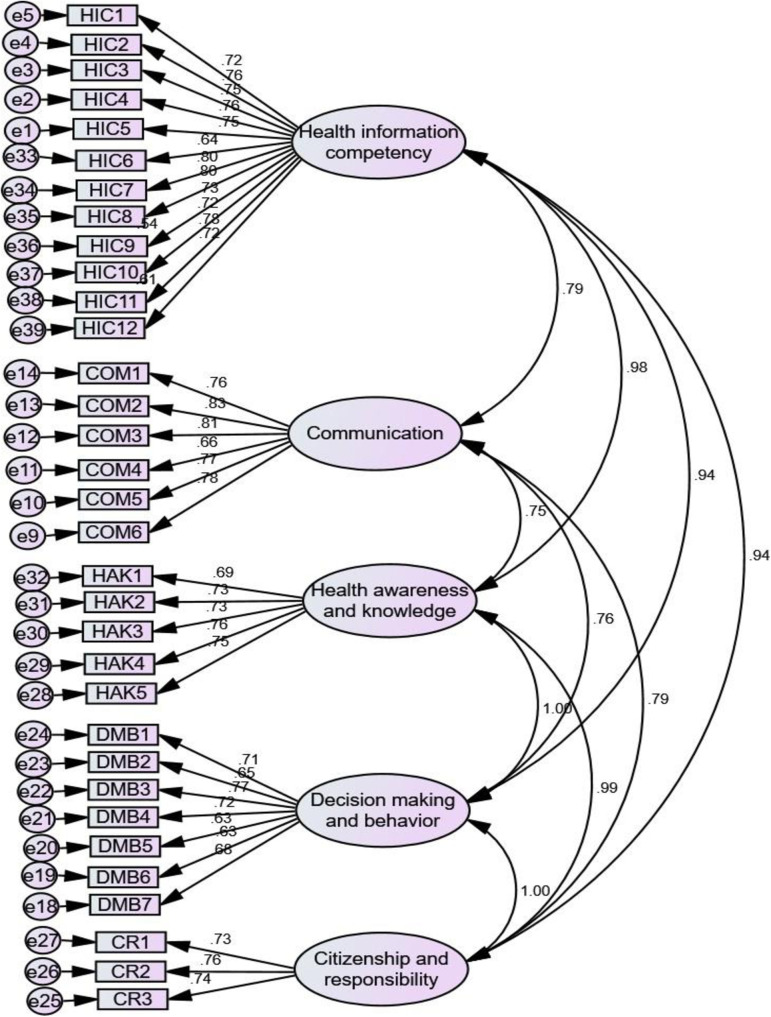
Amharic version.

### The scale’s content, cognitive, and usability standards evaluation

The results of the second stage of the study, which evaluated the scale’s content, cognitive, and usability standards through experts’ reviews and evaluations, cognitive interviews, and a pilot survey, are presented below.

Following the qualitative evaluation of the scale’s content standard by seven experts, one item was removed as it lacked significant distinctiveness (as it addressed almost the same issue as another item), and several of the remaining items were rewritten and improved based on their feedback. Quantitative evaluations of the content validity of the scale (the necessity and relevance of the items of the scale) by 11 experts and the results of the calculated CVR and CVI led to the removal of two of the items, as they scored unacceptable values. The CVR and CVI of the remaining items ranged from 0.64 to 1 and 0.82 to 1, respectively, and the total CVR and CVI scores of the scale were 0.85 and 0.96 respectively, for both versions of the scale, indicating good results.

In cognitive interviews with adolescents about their thoughts while reading the questions, in many cases, the participants stated appreciating the importance of the questions for adolescents, learning or noting important issues from the questions, and engaging in self-evaluation in response to the issues raised. In many cases, the participants stated that none of the questions were confusing. They stated that they answered the questions with understanding, and they also indicated a preference for the Likert-scale format used in this study (strongly disagree, disagree, agree, and strongly agree). However, some participants complained that some statements looked similar and thus were confusing. Based on the feedback, some statements were modified and clarified, and two statements were rewritten and combined into one inclusive statement.

The pilot survey (conducted to evaluate widely the scale’s format, identify items with unacceptable missing values, ceiling effect or floor effect, and to check the internal consistency of the items) result indicated no significant issues necessitating revisions to the format, nor were there significant missing values. However, unacceptable ceiling effect was observed for four items and floor effect for two items. Thus, these statements were modified and reduced to three statements without missing their core ideas. The pilot survey data showed good internal consistency for the scale as well as its subscales. For the entire scale, it was 0.978 and 0.969 and for the subscales it ranged from 0.901 to 0.965 and from 0.821 to 0.950 for the Afaan Oromo version and Amharic version, respectively. Thus, after all the aforesaid modifications were made, the items of the scale finally reduced to 33 (See S4 File).

### Validity and reliability reassurance

This section presents the results of the final stage cross-sectional survey, conducted to further evaluate the scale’s (construct) validity and reliability using CFA and reliability tests, respectively. The values of the statistical measures considered in CFA, namely, χ2/df, CFI, TLI, IFI, RMSEA, and SRMR were initially 1.775, 0.955, 0.951, 0.955, 0.047, and 0.047 respectively, for the Afaan Oromo version, and they were improved to 1.412, 0.976, 0.974, 0.976, 0.034, and 0.031 respectively after creating covariance of error terms (e11&e12, e11&e14, and e18&e19) based on modification indices > 20 [69,83,84]. For the Amharic version, the corresponding values were 1.400, 0.965, 0.962, 0.965, 0.039, and 0.036 respectively. Thus, the obtained results demonstrated strong fit statistics [71,73–76]. However, χ2 was significant (p < 0.05) for both versions, which is a common occurrence in this type of analysis often due to sample size [39,74,85,86]. The items standardized factor loadings were also above 0.60 (it ranges from 0.650 to 0.869 for the Afaan Oromo version and from 0.632 to 0.828 for the Amharic version) indicating good validity [87] (See Figs 1 and 2). As well, the reliability tests also provided good internal consistency for both versions. The internal consistency tests yielded Cronbach’s alpha coefficient of 0.973 and 0.970 for the entire scale and it ranged from 0.850 to 0.937 and from 0.783 to 0.937 for the subscales for the Afaan Oromo version and Amharic version, respectively. And the time stability test (ICC) was 0.715 and 0.712 for the entire scale and it ranged from 0.723 to 0.787 and from 0.708 to 0.794 for the subscales, for the Afaan Oromo version and Amharic version, respectively (See Table 2 and S5 and S6 Files).

**Table 2:** Cronbach’s α coefficient and intraclass correlation coefficient (ICC) of the scale and its subscales.

Dimension	Number of items	Afaan Oromo version		Amharic version	
		Cronbach’s α coefficient (n = 350)	ICC (n = 32)	Cronbach’s α coefficient (n = 261)	ICC (n = 32)
Health information competency	12	0.937	0.731	0.937	0. 752
Communication	6	0.879	0.787	0.895	0. 708
Health awareness and knowledge	5	0.876	0.745	0.851	0. 777
Decision-making and behavior	7	0.908	0.723	0.861	0. 755
Citizenship and responsibility	3	0.850	0.777	0.783	0. 794
Total	33	0.973	0.715	0.970	0.712

## Discussion

This study aimed to develop and validate an AHLS using a mixed methods approach–the recommended approach for this type of study (tool development and validation) [47,88]–to support or guide interventions and measurements. The development and validation of the scale went through three stages: a) generating concepts, categories/dimensions, and items, using in-depth interviews and FGDs as methods of data collection, and designing the scale based on the generated concepts, categories/dimensions, and items, b) evaluating the content, cognitive, and usability standards of the scale using expert reviews and evaluations, cognitive interviews with adolescents, and a pilot survey on a sample of adolescents, and making modifications based on the evaluations, and c) further evaluating the validity and reliability of the scale by collecting data from a large, randomly selected sample of adolescents, using CFA and reliability tests. The final validated scale consisted of 33 items with five major dimensions, namely health information competency (12 items encompassing abilities to access, understand, appraise, and apply health information), communication (6 items), health awareness and knowledge (5 items), decision-making and behavior (7 items), and citizenship and responsibility (3 items), reflecting the multifaceted nature of HL [2,8,89].

The current HL scale embodies the main HL perspectives (healthcare and health promotion) and the core HL abilities (accessing, understanding, appraising, and applying health information) identified by experts in the field [1,2]. The current scale also aligns with the study of Bröder et al. [8], which considers knowledge, health information-related competencies, communication and interaction, as well as citizenship as core aspects of HL in young people. Consistent with the current work, other studies have also noted decision making and behavior as basic features of HL, alongside the health information-related competencies such as accessing, understanding, and appraising and judging health information [90,91].

As well, various adolescent HL tools developed and validated in different countries highlight the multidimensionality of adolescent HL, in line with the current scale, but with varying conceptualizations, patterns of factors, and levels of complexity. For instance, the Multidimensional Measure of Adolescents Health Literacy [34] comprises 24 items with six dimensions, namely, patient-provider encounter, interaction with the health care system, rights and responsibilities, confidence in health information from personal source, confidence in health information from media source, and health information-seeking competency using Internet. Likewise, the Health Literacy Assessment Scale for Adolescents [36] involves 15 items under three dimensions or subscales: communication scale, confusion scale, and functional health literacy scale. Moreover, the Health Literacy Measure for Adolescents [40] has 44 items with eight dimensions, namely, accessing, reading, understanding, appraisal, use, communication, self-efficacy, and numeracy. As well, Measurement of Health Literacy Among Adolescents Questionnaire [39] encompasses 43 items divided into five dimensions: Dealing with health-related information, interactions and communication skills, attitudes toward one’s own health and health information, health-related knowledge, and support for health-related issues by social agents. In addition, some statements within tools such as [39,40,67,92] have similarities with the items on the current scale, with different conceptualizations or expressions.

The validity test of the current scale, based on goodness-of-fit indices, namely, CFI, TLI, IFI, RMSEA, and SRMR, as well as χ2/df, yielded strong results for both versions [71,73–76]. For both versions p-value for χ2 was < 0.05, but this is expected as χ2 used in CFA is usually sensitive to sample size [39,74,85,86]. The entire scale and subscales also had high levels of internal reliability but with some variations between the two versions and among the dimensions of the scale. For both versions, the Cronbach’s alpha coefficient for the entire scale was, in general, excellent [59,77–79]. For the Afaan Oromo version subscales, it ranged from 0.850 to 0.937; while for the Amharic version subscales, it ranged from 0.783 to 0.937. In both cases, the lowest value is obtained for ‘citizenship and responsibility’ dimension and the highest value is obtained for ‘health information competency’ dimension. The low values for the ‘citizenship and responsibility’ dimension may be associated with the small number of items for this dimension [93], as well as with the nature of the sample particularly in the case of the Amharic version, as the respondents were mostly from the urban background (homogeneity) for this version [39,94]. The time stability test for the entire scale and its sub-scales also provided good result for both versions [58,80,82].

This work has crucial implications for interventions and future research. The identified dimensions of the scale demonstrate the HL knowledge, abilities, skills, and related qualities that adolescents need to take control of their health and health behaviors and to contribute to the health and wellbeing of their families and communities. Thus, this scale is essential for designing effective interventions to promote and foster HL in adolescents as well as to evaluate it. The scale has a great importance for researchers interested in adolescents’ HL in particular and HL in general, as it can also be used for other populations making necessary modifications, including adding extra items if needed. However, as this was the first attempt to develop a HL scale for adolescents in Ethiopia, and as a tool validation is an ongoing process, further studies are needed with adolescents of different age groups and in different contexts to ensure the scale’s validity and reliability and enhance its applicability and generalizability.

### Strengths and limitations

Measurement of HL in adolescents is vital to have a deeper insight into their HL situation, including the influencing factors, and to design effective interventions to promote HL and healthy lifestyles in this age group. Lack of a contextualized and appropriate measurement tool is one of the challenges to attain this goal in developing countries like Ethiopia. This study is the first attempt, in this context, to develop an adolescent HL scale grounded in qualitative data and to validate it using both qualitative and quantitative approaches. This scale fills critical gaps and provides a foundation for broader measurement of HL in adolescents, for the design of effective interventions, and for the progress of HL in Ethiopia as well as in other related contexts. However, this study is not without limitations. First of all, the study was limited to school contexts. Thus, although the sample was diverse, representing adolescents from various sociodemographic backgrounds, it may not be representative of out-of-school adolescents. Moreover, in this study, convergent validity testing was not conducted to determine the degree to which the current scale correlates with another known, valid instrument measuring the same issue. Thus, future studies need to fill these gaps.

## Conclusion

The study developed and validated an adolescent HL scale using a mixed methods approach within the socio-cultural context of Ethiopia, addressing a critical gap in this area. This scale is multidimensional, encompassing HL knowledge, skills, abilities, and related attributes or qualities essential for adolescents today. The scale can be adapted and used with populations beyond adolescents and in other contexts in future studies. It is also vital for designing effective strategies aimed at improving HL in adolescents.

## Supporting information

S1 File Research team and reflexivity, study design, and analysis related issues on the first stage of the study. (DOCX)

S2 File Sociodemographic characteristics of the participants (from stage I to stage III). (DOCX)

S3 File AHLS (Adolescent HL scale), the first draft. (DOCX)

S4 File AHLS (Adolescent HL scale), the final draft. (DOCX)

S5 File Psychometric properties of the scale – Afaan Oromo version. (DOCX)

S6 File Psychometric properties of the scale – Amharic version. (DOCX)
