# Supplementary material for: Development and validation of an adolescent health literacy scale in Ethiopia: A mixed methods approach
Source: PLoS One. 2025 Aug 8;20(8):e0329184. doi: 10.1371/journal.pone.0329184 (PMC12334042; doi:10.1371/journal.pone.0329184)
Supplement: S1 File — (DOCX) [file pone.0329184.s001.docx]

# S1 Supplementary file: Research team and reflexivity, Study design, and Analysis related issues on the first stage of the study

All of the authors are academic staff at Jimma University and they are all male. The first author holds expertise in Sociology (Health and Wellbeing) and Public Health (Health Communication and Health Behavior). The second author’s specialization lies in Sociology and Social Anthropology, and the third author’s focus is on Health Education, Health Promotion, and Evidence-based Healthcare.

The first stage of the study utilized in-depth interviews and focus group discussions (FGDs), guided by a grounded theory, to explore the adolescents’ HL perspectives and experiences, and to generate concepts, categories, and items for the scale design. The study focused on school adolescents. Purposive sampling with a maximal variation sampling strategy was used to ensure diversity among participants in terms of sex, age, grade/class level, parental socioeconomic factors, and other relevant characteristics.

The interviews and FGDs were conducted by the first author. He took adequate time to build trust and good relationships with participants before the start of the data collection. He explained the study’s aim to the potential participants and obtained informed consent/assent from those who were willing to participate. Of the invited participants, three adolescents (one male and two female) refused to participate without providing a reason, and one female adolescent withdrew after beginning participation due to lack of interest to talk on the issue.

The study used a data collection guide, which was constructed based on the purpose of the study and insights gained from HL literature, and then tested for clarity and appropriateness. During the interviews and FGDs, subsequently, the questions the participants were asked include: Are you interested in learning about health? What does being health literate or health literacy mean for you? Are you a health literate person? What abilities and skills do you need to be a health literate person? Do you seek health information? From where do you often get health information? Do you understand the health information you acquire from different sources? If the health information you acquired is unclear, what do you do? Do you question the accuracy of the health information you acquire from different sources? How do you ensure the credibility of the health information you access? What do you do with the health information you acquire? Do you use it in your daily life?

The interviews and FGDs were carried out in school compounds, at comfortable places (either in free class rooms or outside the class rooms) (face-to-face) in either Afaan Oromo or Amharic, based on the participants’ language preferences. The responses of the participants were rephrased, summarized, and reflected back to them to avoid misunderstandings and misinterpretations. Sampling and data collection continued until the achievement of data saturation (the saturation of information and concepts). In total, 86 male and female adolescents, aged 14-19 years old participated in the study (the first stage). Forty seven of them participated in in-depth interviews (22 males & 25 females), and the remaining 39 participated in six separate FGDs (19 males & 20 females). Almost all of the conversations were audio recorded based on the participants’ permissions, and the duration of the conversations varied from about 24 to 75 minutes. Fieldnotes were also recorded during the data collection process to enrich the study.

Following completion, each interview and FGD was translated word by word. Then, the translated text was sorted and segmented; important quotes and segments were identified; the segments were coded; codes were categorized and grouped into broader dimensions using Atlas.ti version 7.5.18 software. This all were then revised by the team members. The researcher approached the data with an open mind over the course of the study, while acknowledging the influence of key concepts form HL literature he was reading.
