# Supplementary material for: Development and validation of an adolescent health literacy scale in Ethiopia: A mixed methods approach
Source: PLoS One. 2025 Aug 8;20(8):e0329184. doi: 10.1371/journal.pone.0329184 (PMC12334042; doi:10.1371/journal.pone.0329184)
Supplement: S2 File — (DOCX) [file pone.0329184.s002.docx]

# S2 Supplementary file: Sociodemographic characteristics of the participants (from stage I to stage III)

| Variable | Category | Frequency (Percentage) |
| --- | --- | --- |
| **Sex**  (In the age range of 14 to 19 with mean age of 16.75 ± 1.28 years) | Male | 391 (45.36%) |
|  | Female | 471 (54.64%) |
|  | Total | 862(100%) |
| School type (grade 9 to 12) | Public | 626 (72.62%) |
|  | Private | 236 (27.38%) |
| **Religion** | Islam | 415 (48.14%) |
|  | Orthodox | 247 (28.65%) |
|  | Protestant | 178 (20.65%) |
|  | Other | 22 (2.55%) |
| **Origin** (primary school background) | Urban | 612 (71.00%) |
|  | Rural | 250 (29.00%) |
| **Father’s education** | Don’t know | 146 (16.94%) |
|  | Unable to read and write | 107 (12.41%) |
|  | Primary school | 224 (25.99%) |
|  | Secondary school | 180 (20.88%) |
|  | Diploma | 62 (7.19%) |
|  | Degree/above | 143 (16.59%) |
| **Mother’s education** | Don’t know | 114 (13.23%) |
|  | Unable to read and write | 145 (16.82%) |
|  | Primary school | 264 (30.63%) |
|  | Secondary school | 170 (19.72%) |
|  | Diploma | 71 (8.24%) |
|  | Degree/above | 98 (11.36%) |
| **Father’s occupation** | Merchant | 252 (29.23%) |
|  | Government employee | 219 (25.41%) |
|  | Framer | 258 (29.93%) |
|  | Other | 133 (15.43%) |
| **Mother’s occupation** | Merchant | 209 (24.25%) |
|  | Government employee | 155 (17.98%) |
|  | Farmer | 96 (11.14%) |
|  | Housewife | 360 (41.76%) |
|  | Other | 42 (4.87%) |
