# Supplementary material for: Development and validation of an adolescent health literacy scale in Ethiopia: A mixed methods approach
Source: PLoS One. 2025 Aug 8;20(8):e0329184. doi: 10.1371/journal.pone.0329184 (PMC12334042; doi:10.1371/journal.pone.0329184)
Supplement: S3 File — (DOCX) [file pone.0329184.s003.docx]

# S3 Supplementary file: AHLS (Adolescent HL scale), the first draft

1. You know where and how to find the health information you need.
2. You are able to access health information you need from various sources.
3. You can access information on reproductive health (RH), including issues related to adolescence and sexually transmitted diseases/infections (STDs/STIs).
4. You can access information on the risks of unhealthy behaviors such as smoking, alcohol use, and addicting substances.
5. You are able to access information about the benefits of healthy eating or diets, good sleep, and regular physical activity.
6. You can easily understand the health information you obtain from diverse sources.
7. You can easily read and understand health information from various sources, including online and print materials.
8. You can easily read and/or understand healthcare provider and pharmacist instructions and prescriptions.
9. You can judge quality of the health information you obtain from various sources.
10. You can compare, contrast, and resolve conflicting health information from different sources.
11. You actively engage in accessing credible health information to maintain your health.
12. You apply credible health information you access from various sources in your everyday life.
13. You can freely ask a trusted individual to clarify any unclear health information.
14. You can openly discuss your health concerns with your parents.
15. You can openly discuss your RH concerns with your parents.
16. You can openly discuss your health concerns with others who you believe have knowledge of or experience with the issue.
17. You can freely discuss your RH concerns with others who you believe have knowledge of or experience with the issue.
18. You ask for assistance when uncertain about the quality of health information you get.
19. You openly discuss your health and related concerns with your parents
20. If you experience health problems, like STDs/STIs symptoms, you ask for help from your parents.
21. You openly discuss your health and related concerns freely with others who you believe have knowledge of or experience with the issue.
22. You can understand/judge how your own actions/behaviors affect you and others.
23. You believe you have sufficient information to maintain your health.
24. You believe you have sufficient knowledge to maintain your health.
25. You think that you have enough knowledge about RH, including issues related to adolescence and STDs/STIs.
26. You are well informed about the health benefits of healthy diet, sleeping well, and physical activity.
27. You are well informed about the health warnings of behaviors like smoking, alcohol use and other drug addictions.
28. You are well informed about the behavioral risk factors for overweight, high cholesterol, diabetes, cancer, and cardiovascular diseases.
29. You have enough information about why health screenings, including breast, vaginal, blood sugar, and blood cholesterol tests, as well as vaccinations are needed.
30. You can judge when and where you should seek health services.
31. You can decide what you should do and should not do to maintain your health.
32. You can make informed decisions to improve and maintain your health based on information you obtain from various sources.
33. You take care of your health every day or consistently based on the health information you obtain.
34. You avoid substances like cigarette, alcohol, and other substances that are risky to your health.
35. You protect yourself from STDs/STIs and unplanned pregnancy.
36. If you experience health problems, like STDs/STIs symptoms, you promptly seek for help from a health professional.
37. You accurately follow health instruction you obtain from healthcare providers and pharmacists.
38. You believe that your health knowledge could be useful to your family members, friends, and others.
39. You share your health knowledge with friends to help them avoid risky behaviors (addiction and others), and adopt healthy habits.
40. You actively engage in health promotion activities like sanitation practices and health awareness campaigns within your school and community.
