# Supplementary material for: Development and validation of an adolescent health literacy scale in Ethiopia: A mixed methods approach
Source: PLoS One. 2025 Aug 8;20(8):e0329184. doi: 10.1371/journal.pone.0329184 (PMC12334042; doi:10.1371/journal.pone.0329184)
Supplement: S4 File — (DOCX) [file pone.0329184.s004.docx]

# S4 Supplementary file: AHLS (Adolescent HL scale), the final draft

1. **HIC1-**You know where and how to access the health information you need.
2. **HIC2-**You are able to access health information you need from various sources.
3. **HIC3-**You can access information on reproductive health (RH), including issues related to adolescence and sexually transmitted diseases/infections (STDs/STIs).
4. **HIC4-**You can find information on why you should avoid unhealthy behaviors such as smoking, alcohol use, and other addictive substances.
5. **HIC5-**You can access information about the health benefits of healthy eating or diets, good sleep, and regular physical activity.
6. **HIC6-**You can easily understand the health information you obtain from various sources.
7. **HIC7-**You can easily read and understand health information from various sources, including online and print materials.
8. **HIC8-**You can easily read and/or understand healthcare provider and/or pharmacist instructions and prescriptions.
9. **HIC9-**You can judge the quality of health information you obtain from various sources.
10. **HIC10-**You can compare, contrast, and resolve conflicting health information from different sources.
11. **HIC11**-You actively engage in seeking and accessing creditable health information to maintain and improve your health.
12. **HIC12-**You apply credible health information you access from various sources in your everyday life.
13. **COM1-**You can freely consult a trusted individual for assistance with any unclear or questionable health information or related issue.
14. **COM2-**You can openly discuss any health concerns you have, including those related to adolescence and RH, with your parents.
15. **COM3-**You can openly discuss any health concerns you have, including issues related to adolescence and RH, with others whom you believe have knowledge of or experience in the matter.
16. **COM4-**Whenever you come across unclear or questionable health information, you freely ask a trusted individual for clarification or assistance.
17. **COM5-**Whenver you face any health problem, including RH-related problems or STDs/STIs symptoms, you openly talk about your concerns with your parents.
18. **COM6-**Whenever you experience any health problem, including RH issues or symptoms of STDs/STIs, you openly discuss your concerns with others who you believe have knowledge or experience in the issue.
19. **HAK1-**You are aware of that or how your own actions and behaviors affect you and others.
20. **HAK2-**You have adequate information for your age regarding healthy and unhealthy behaviors.
21. **HAK3-**You have adequate information and knowledge for your age regarding RH, including issues related to adolescence and STDs/STIs.
22. **HAK4-**You are well informed about the behavioral risk factors for non-communicable disease, such as chronic respiratory diseases, cardiovascular diseases, cancer, and diabetes, as well as mental health issues.
23. **HAK5-**You are well informed about the need for health screenings, like breast and pelvic tests for females and blood sugar and cholesterol exams or general checkups, as well as vaccinations.
24. **DMB1-**You can usually judge when and where you should seek health services.
25. **DMB2-**You can decide what to do and not to do to maintain your health based on information you obtain from various sources.
26. **DMB3-**You take care of or prioritize your health every day, based on information you have obtained, regardless of the conditions.
27. **DMB4-**You avoid substances like cigarette, alcohol, and other substances as well as too much sweet diet that are not good for your health.
28. **DMB5-**You protect yourself from unhealthy relationships, unplanned pregnancy, and STDs/STIs.
29. **DMB6-**Whenever you experience any health problem, including RH related problems or STDs/STIs symptoms, you promptly seek help from a health professional.
30. **DMB7-**You accurately follow the health advice, instructions, and directions you receive from a healthcare provider and/or a pharmacist.
31. **CR1-**You believe that your health knowledge and understanding are valuable to your family members, friends, and others.
32. **CR2-**You share your health knowledge with friends and help them avoid risky behaviors, such as addiction, and adopt healthy habits.
33. **CR3-**You actively participate in health promotion efforts, such as sanitation activities, health awareness campaigns or clubs, and other initiatives within your school and community.
