# Supplementary material for: Development and validation of an adolescent health literacy scale in Ethiopia: A mixed methods approach
Source: PLoS One. 2025 Aug 8;20(8):e0329184. doi: 10.1371/journal.pone.0329184 (PMC12334042; doi:10.1371/journal.pone.0329184)
Supplement: S5 File — (DOCX) [file pone.0329184.s005.docx]

# S5 Supplementary file: Psychometric properties of the scale - Afaan Oromo version

**S5.1. Standardized Regression Weights: (Group number 1 - Default model)**

|  |  |  | Estimate |
| --- | --- | --- | --- |
| Q8HIC8 | <--- | F1 | .762 |
| Q9HIC9 | <--- | F1 | .790 |
| Q10HIC10 | <--- | F1 | .718 |
| Q11HIC11 | <--- | F1 | .748 |
| Q12HIC12 | <--- | F1 | .730 |
| Q13COM1 | <--- | F2 | .685 |
| Q14COM2 | <--- | F2 | .703 |
| Q15COM3 | <--- | F2 | .820 |
| Q16COM4 | <--- | F2 | .705 |
| Q17COM5 | <--- | F2 | .650 |
| Q18COM6 | <--- | F2 | .829 |
| Q24DMB1 | <--- | F4 | .770 |
| Q25DMB2 | <--- | F4 | .787 |
| Q26DMB3 | <--- | F4 | .748 |
| Q27DMB4 | <--- | F4 | .741 |
| Q28DMB5 | <--- | F4 | .832 |
| Q29DMB6 | <--- | F4 | .683 |
| Q30DMB7 | <--- | F4 | .806 |
| Q31CR1 | <--- | F5 | .743 |
| Q32CR2 | <--- | F5 | .824 |
| Q33CR3 | <--- | F5 | .869 |
| Q19HAK1 | <--- | F3 | .770 |
| Q20HAK2 | <--- | F3 | .769 |
| Q21HAK3 | <--- | F3 | .795 |
| Q22HAK4 | <--- | F3 | .685 |
| Q23HAK5 | <--- | F3 | .808 |
| Q7HIC7 | <--- | F1 | .801 |
| Q6HIC6 | <--- | F1 | .780 |
| Q5HIC5 | <--- | F1 | .730 |
| Q4HIC4 | <--- | F1 | .696 |
| Q3HIC3 | <--- | F1 | .696 |
| Q2HIC2 | <--- | F1 | .665 |
| Q1HIC1 | <--- | F1 | .816 |

**S5.2. Reliability (Cronbach's alpha coefficient)**

**Scale: ALL VARIABLES**

| **Case Processing Summary** | | | |
| --- | --- | --- | --- |
|  | | N | % |
| Cases | Valid | 350 | 100.0 |
|  | Excluded^a^ | 0 | .0 |
|  | Total | 350 | 100.0 |
| a. Listwise deletion based on all variables in the procedure. | | | |

| **Reliability Statistics** | |
| --- | --- |
| Cronbach's Alpha | N of Items |
| .973 | 33 |

**Health information competency**

| Reliability Statistics | |
| --- | --- |
| Cronbach's Alpha | N of Items |
| .937 | 12 |

| Item-Total Statistics | | | | |
| --- | --- | --- | --- | --- |
|  | Scale Mean if Item Deleted | Scale Variance if Item Deleted | Corrected Item-Total Correlation | Cronbach's Alpha if Item Deleted |
| HIC1-You know where and how to access the health information and services you need. | 28.45 | 35.056 | .775 | .930 |
| HIC2-You are able to access health information you need from various sources. | 28.41 | 35.927 | .653 | .934 |
| HIC3-You can access information on reproductive health (RH), including issues related to adolescence and sexually transmitted diseases/infections (STDs/STIs). | 28.47 | 35.436 | .687 | .933 |
| HIC4-You can find information on why you should avoid unhealthy behaviors such as smoking, alcohol use, and other addictive substances. | 28.45 | 35.102 | .678 | .933 |
| HIC5-You can access information about the health benefits of healthy eating or diets, good sleep, and regular physical activity. | 28.41 | 35.256 | .707 | .932 |
| HIC6-You can understand the health information you obtain from various sources. | 28.43 | 35.111 | .742 | .931 |
| HIC7-You can easily read and understand health information from various sources, including online and print materials. | 28.37 | 34.808 | .770 | .930 |
| HIC8-You can easily read and/or understand healthcare provider and/or pharmacist instructions and prescriptions. | 28.60 | 34.448 | .742 | .931 |
| HIC9-You can judge the quality of health information you obtain from various sources. | 28.76 | 35.514 | .763 | .930 |
| HIC10-You can compare, contrast, and resolve conflicting health information from different sources. | 28.65 | 34.870 | .693 | .933 |
| HIC11-You actively engage in seeking and accessing credible health information to maintain and improve your health. | 28.83 | 34.769 | .727 | .931 |
| HIC12-You apply credible health information you accessed from various sources in your everyday life. | 28.66 | 35.875 | .702 | .932 |

**Communication**

| Reliability Statistics | |
| --- | --- |
| Cronbach's Alpha | N of Items |
| .879 | 6 |

| Item-Total Statistics | | | | |
| --- | --- | --- | --- | --- |
|  | Scale Mean if Item Deleted | Scale Variance if Item Deleted | Corrected Item-Total Correlation | Cronbach's Alpha if Item Deleted |
| COM1-You can freely consult a trusted individual for clarification or assistance with any unclear or questionable health information or other issue. | 12.90 | 8.535 | .624 | .868 |
| COM2-You can openly discuss any health concerns you have, including those related to adolescence and RH, with your parents. | 13.25 | 8.070 | .654 | .864 |
| COM3-You can openly discuss any health concerns you have, including issues related to adolescence and RH, with others whom you believe have knowledge of or experience in the matter. | 13.11 | 7.927 | .786 | .841 |
| COM4-Whenever you come across unclear or questionable health information, you freely ask a trusted individual for clarification or assistance. | 13.02 | 8.263 | .640 | .866 |
| COM5-Whenever you face any health problem, including RH-related problems or STDs/STIs symptoms, you openly talk about your concerns with your parents. | 13.24 | 8.574 | .605 | .871 |
| COM6-Whenever you experience any health problem, including RH issues or symptoms of STDs/STIs, you openly discuss your concerns with others who you believe have knowledge or experience in the issue. | 13.11 | 7.656 | .815 | .835 |

**Health awareness and knowledge**

| Reliability Statistics | |
| --- | --- |
| Cronbach's Alpha | N of Items |
| .876 | 5 |

| Item-Total Statistics | | | | |
| --- | --- | --- | --- | --- |
|  | Scale Mean if Item Deleted | Scale Variance if Item Deleted | Corrected Item-Total Correlation | Cronbach's Alpha if Item Deleted |
| HAK1-You are aware of that or how your own actions and behaviors affect you and others. | 9.60 | 5.054 | .682 | .855 |
| HAK2-You have you adequate health information for your age regarding healthy and unhealthy behaviors. | 10.07 | 5.073 | .740 | .841 |
| HAK3-You have adequate information and knowledge for your age regarding RH, including puberty, pregnancy, and STDs/STIs. | 9.92 | 4.775 | .737 | .842 |
| HAK4-You are well informed about the behavioral risk factors for noncommunicable disease, such as chronic respiratory diseases, cardiovascular diseases, cancer, and diabetes, as well as mental health issues. | 9.97 | 5.555 | .638 | .865 |
| HAK5-You are well informed about the need for health screenings, like breast and pelvic tests for females and blood sugar and cholesterol tests or general health checkups, as well as vaccinations. | 9.92 | 4.893 | .738 | .841 |

**Decision making and behavior**

| Reliability Statistics | |
| --- | --- |
| Cronbach's Alpha | N of Items |
| .908 | 7 |

| Item-Total Statistics | | | | |
| --- | --- | --- | --- | --- |
|  | Scale Mean if Item Deleted | Scale Variance if Item Deleted | Corrected Item-Total Correlation | Cronbach's Alpha if Item Deleted |
| DMB1-You can judge when and where you should seek health services. | 15.96 | 10.723 | .749 | .891 |
| DMB2-You can decide what to do and not to do to stay healthy or to protect your health, based on information you get from various sources. | 15.73 | 10.455 | .770 | .889 |
| DMB3-You take care of or prioritize your health every day, based information you have obtained, regardless of the circumstance. | 15.92 | 10.532 | .714 | .895 |
| DMB4-You avoid substances like cigarette, alcohol, and other substances as well as too much sweet diet that are not good for your health. | 15.71 | 10.577 | .695 | .898 |
| DMB5-You protect yourself from unhealthy relationships, unplanned pregnancy, and STDs/STIs. | 15.71 | 10.655 | .771 | .889 |
| DMB6- Whenever you experience any health problem, including RH related problems or STDs/STIs symptoms, you promptly seek help from a health professional. | 15.65 | 10.785 | .631 | .905 |
| DMB7-You accurately follow the health advice, instructions, and directions you receive from a healthcare provider and/or a pharmacist. | 15.76 | 11.060 | .764 | .891 |

**Citizenship and responsibility**

| Reliability Statistics | |
| --- | --- |
| Cronbach's Alpha | N of Items |
| .850 | 3 |

| Item-Total Statistics | | | | |
| --- | --- | --- | --- | --- |
|  | Scale Mean if Item Deleted | Scale Variance if Item Deleted | Corrected Item-Total Correlation | Cronbach's Alpha if Item Deleted |
| CR1-You believe that your health knowledge and understanding are valuable to your family members, friends, and others. | 5.08 | 1.756 | .657 | .847 |
| CR2-You share your health knowledge with friends to help them avoid risky behaviors, such as addiction, and adopt healthy habits. | 5.18 | 1.478 | .746 | .765 |
| CR3-You actively participate in health promotion efforts, such as sanitation activities, health awareness campaigns or clubs, and other initiatives within your school and community. | 5.25 | 1.544 | .761 | .751 |

**S5.3. Test-retest (Intra-class Correlation Coefficients)**

| **Case Processing Summary** | | | |
| --- | --- | --- | --- |
|  | | N | % |
| Cases | Valid | 32 | 100.0 |
|  | Excluded^a^ | 0 | .0 |
|  | Total | 32 | 100.0 |
| a. Listwise deletion based on all variables in the procedure. | | | |

| **Intraclass Correlation Coefficient** | | | | | | | |
| --- | --- | --- | --- | --- | --- | --- | --- |
|  | Intraclass Correlation^b^ | 95% Confidence Interval | | F Test with True Value 0 | | | |
|  |  | Lower Bound | Upper Bound | Value | df1 | df2 | Sig |
| Single Measures | .715^a^ | .615 | .817 | 169.952 | 31 | 2015 | .000 |
| Average Measures | .994 | .991 | .997 | 169.952 | 31 | 2015 | .000 |
| Two-way random effects model where both people effects and measures effects are random. | | | | | | | |
| a. The estimator is the same, whether the interaction effect is present or not. | | | | | | | |
| b. Type A intraclass correlation coefficients using an absolute agreement definition. | | | | | | | |

**Health information competency**

| **Intraclass Correlation Coefficient** | | | | | | | |
| --- | --- | --- | --- | --- | --- | --- | --- |
|  | Intraclass Correlation^b^ | 95% Confidence Interval | | F Test with True Value 0 | | | |
|  |  | Lower Bound | Upper Bound | Value | df1 | df2 | Sig |
| Single Measures | .731^a^ | .630 | .830 | 69.765 | 31 | 713 | .000 |
| Average Measures | .985 | .976 | .992 | 69.765 | 31 | 713 | .000 |
| Two-way random effects model where both people effects and measures effects are random. | | | | | | | |
| a. The estimator is the same, whether the interaction effect is present or not. | | | | | | | |
| b. Type A intraclass correlation coefficients using an absolute agreement definition. | | | | | | | |

**Communication**

| **Intraclass Correlation Coefficient** | | | | | | | |
| --- | --- | --- | --- | --- | --- | --- | --- |
|  | Intraclass Correlation^b^ | 95% Confidence Interval | | F Test with True Value 0 | | | |
|  |  | Lower Bound | Upper Bound | Value | df1 | df2 | Sig |
| Single Measures | .787^a^ | .695 | .870 | 45.778 | 31 | 341 | .000 |
| Average Measures | .978 | .965 | .988 | 45.778 | 31 | 341 | .000 |
| Two-way random effects model where both people effects and measures effects are random. | | | | | | | |
| a. The estimator is the same, whether the interaction effect is present or not. | | | | | | | |
| b. Type A intraclass correlation coefficients using an absolute agreement definition. | | | | | | | |

**Health awareness and knowledge**

| **Intraclass Correlation Coefficient** | | | | | | | |
| --- | --- | --- | --- | --- | --- | --- | --- |
|  | Intraclass Correlation^b^ | 95% Confidence Interval | | F Test with True Value 0 | | | |
|  |  | Lower Bound | Upper Bound | Value | df1 | df2 | Sig |
| Single Measures | .745^a^ | .641 | .843 | 30.933 | 31 | 279 | .000 |
| Average Measures | .967 | .947 | .982 | 30.933 | 31 | 279 | .000 |
| Two-way random effects model where both people effects and measures effects are random. | | | | | | | |
| a. The estimator is the same, whether the interaction effect is present or not. | | | | | | | |
| b. Type A intraclass correlation coefficients using an absolute agreement definition. | | | | | | | |

**Decision making and behavior**

| **Intraclass Correlation Coefficient** | | | | | | | |
| --- | --- | --- | --- | --- | --- | --- | --- |
|  | Intraclass Correlation^b^ | 95% Confidence Interval | | F Test with True Value 0 | | | |
|  |  | Lower Bound | Upper Bound | Value | df1 | df2 | Sig |
| Single Measures | .723^a^ | .618 | .826 | 37.504 | 31 | 403 | .000 |
| Average Measures | .973 | .958 | .985 | 37.504 | 31 | 403 | .000 |
| Two-way random effects model where both people effects and measures effects are random. | | | | | | | |
| a. The estimator is the same, whether the interaction effect is present or not. | | | | | | | |
| b. Type A intraclass correlation coefficients using an absolute agreement definition. | | | | | | | |

**Citizenship and responsibility**

| **Intraclass Correlation Coefficient** | | | | | | | |
| --- | --- | --- | --- | --- | --- | --- | --- |
|  | Intraclass Correlation^b^ | 95% Confidence Interval | | F Test with True Value 0 | | | |
|  |  | Lower Bound | Upper Bound | Value | df1 | df2 | Sig |
| Single Measures | .777^a^ | .672 | .867 | 22.225 | 31 | 155 | .000 |
| Average Measures | .954 | .925 | .975 | 22.225 | 31 | 155 | .000 |
| Two-way random effects model where both people effects and measures effects are random. | | | | | | | |
| a. The estimator is the same, whether the interaction effect is present or not. | | | | | | | |
| b. Type A intraclass correlation coefficients using an absolute agreement definition. | | | | | | | |
