# Supplementary material for: Development and validation of an adolescent health literacy scale in Ethiopia: A mixed methods approach
Source: PLoS One. 2025 Aug 8;20(8):e0329184. doi: 10.1371/journal.pone.0329184 (PMC12334042; doi:10.1371/journal.pone.0329184)
Supplement: S6 File — (DOCX) [file pone.0329184.s006.docx]

# S6 Supplementary file: Psychometric properties of the scale - Amharic version

**S6.1. Standardized Regression Weights: (Group number 1 - Default model)**

|  |  |  | Estimate |
| --- | --- | --- | --- |
| Q5HIC5 | <--- | F1 | .750 |
| Q4HIC4 | <--- | F1 | .761 |
| Q3HIC3 | <--- | F1 | .754 |
| Q2HIC2 | <--- | F1 | .762 |
| Q1HIC1 | <--- | F1 | .719 |
| Q18COM6 | <--- | F2 | .784 |
| Q17COM5 | <--- | F2 | .775 |
| Q16COM4 | <--- | F2 | .664 |
| Q15COM3 | <--- | F2 | .805 |
| Q14COM2 | <--- | F2 | .828 |
| Q13COM1 | <--- | F2 | .762 |
| Q30DMB7 | <--- | F4 | .681 |
| Q29DMB6 | <--- | F4 | .632 |
| Q28DMB5 | <--- | F4 | .634 |
| Q27DMB4 | <--- | F4 | .722 |
| Q26DMB3 | <--- | F4 | .775 |
| Q25DMB2 | <--- | F4 | .646 |
| Q24DMB1 | <--- | F4 | .706 |
| Q33CR3 | <--- | F5 | .745 |
| Q32CR2 | <--- | F5 | .758 |
| Q31CR1 | <--- | F5 | .727 |
| Q23HAK5 | <--- | F3 | .748 |
| Q22HAK4 | <--- | F3 | .761 |
| Q21HAK3 | <--- | F3 | .729 |
| Q20HAK2 | <--- | F3 | .729 |
| Q19HAK1 | <--- | F3 | .690 |
| Q6HIC6 | <--- | F1 | .642 |
| Q7HIC7 | <--- | F1 | .802 |
| Q8HIC8 | <--- | F1 | .804 |
| Q9HIC9 | <--- | F1 | .733 |
| Q10HIC10 | <--- | F1 | .719 |
| Q11HIC11 | <--- | F1 | .779 |
| Q12HIC12 | <--- | F1 | .722 |

**S6.2. Reliability (Cronbach's alpha coefficient)**

**Scale: ALL VARIABLES**

| **Case Processing Summary** | | | |
| --- | --- | --- | --- |
|  | | N | % |
| Cases | Valid | 261 | 100.0 |
|  | Excluded^a^ | 0 | .0 |
|  | Total | 261 | 100.0 |
| a. Listwise deletion based on all variables in the procedure. | | | |

| Reliability Statistics | |
| --- | --- |
| Cronbach's Alpha | N of Items |
| .970 | 33 |

**Health information competency**

| Reliability Statistics | |
| --- | --- |
| Cronbach's Alpha | N of Items |
| .937 | 12 |

| Item-Total Statistics | | | | |
| --- | --- | --- | --- | --- |
|  | Scale Mean if Item Deleted | Scale Variance if Item Deleted | Corrected Item-Total Correlation | Cronbach's Alpha if Item Deleted |
| HIC1-You know where and how to access the health information you need. | 29.40 | 39.826 | .703 | .933 |
| HIC2-You are able to access health information you need from various sources. | 29.31 | 40.546 | .745 | .931 |
| HIC3-You can access information on reproductive health (RH), including issues related to adolescence and sexually transmitted diseases/infections (STDs/STIs). | 29.37 | 39.934 | .729 | .932 |
| HIC4-You can find information on why you should avoid unhealthy behaviors such as smoking, alcohol use, and other addictive substances. | 29.45 | 39.941 | .737 | .931 |
| HIC5-You can access information about the health benefits of healthy eating or diets, good sleep, and regular physical activity. | 29.33 | 40.529 | .715 | .932 |
| HIC6-You can understand the health information you obtain from various sources. | 29.37 | 41.218 | .616 | .936 |
| HIC7-You can easily read and understand health information from various sources, including online and print materials. | 29.36 | 39.915 | .776 | .930 |
| HIC8-You can easily read and/or understand healthcare provider and/or pharmacist instructions and prescriptions. | 29.63 | 39.465 | .758 | .931 |
| HIC9-You can judge the quality of health information you obtain from various sources. | 29.57 | 39.770 | .716 | .932 |
| HIC10-You can compare, contrast, and resolve conflicting health information from different sources. | 29.62 | 40.137 | .688 | .933 |
| HIC11-You actively engage in seeking and accessing creditable health information to maintain and improve your health. | 29.58 | 39.114 | .750 | .931 |
| HIC12-You apply credible health information you accessed from various sources in your everyday life. | 29.54 | 39.926 | .713 | .932 |

**Communication**

| Reliability Statistics | |
| --- | --- |
| Cronbach's Alpha | N of Items |
| .895 | 6 |

| Item-Total Statistics | | | | |
| --- | --- | --- | --- | --- |
|  | Scale Mean if Item Deleted | Scale Variance if Item Deleted | Corrected Item-Total Correlation | Cronbach's Alpha if Item Deleted |
| COM1-You can freely consult a trusted individual for assistance with any unclear or questionable health information or other issue. | 13.23 | 9.741 | .717 | .877 |
| COM2-You can openly discuss any health concerns you have, including those related to adolescence and RH, with your parents. | 13.57 | 9.077 | .769 | .868 |
| COM3-You can openly discuss any health concerns you have, including issues related to adolescence and RH, with others whom you believe have knowledge of or experience in the matter. | 13.25 | 9.534 | .763 | .870 |
| COM4-Whenever you come across unclear or questionable health information, you freely ask a trusted individual for clarification or assistance. | 13.41 | 9.658 | .616 | .892 |
| COM5-Whenver you face any health problem, including RH-related problems or STDs/STIs symptoms, you openly talk about your concerns with your parents. | 13.57 | 9.030 | .726 | .875 |
| COM6-Whenever you experience any health problem, including RH issues or symptoms of STDs/STIs, you openly discuss your concerns with others who you believe have knowledge or experience in the issue. | 13.30 | 9.478 | .729 | .874 |

**Health awareness and knowledge**

| Reliability Statistics | |
| --- | --- |
| Cronbach's Alpha | N of Items |
| .851 | 5 |

| Item-Total Statistics | | | | |
| --- | --- | --- | --- | --- |
|  | Scale Mean if Item Deleted | Scale Variance if Item Deleted | Corrected Item-Total Correlation | Cronbach's Alpha if Item Deleted |
| HAK1-You are aware of that or how your own actions and behaviors affect you and others. | 10.04 | 6.222 | .602 | .836 |
| HAK2-You have adequate information for your age regarding healthy and unhealthy behaviours. | 10.30 | 5.326 | .695 | .813 |
| HAK3-You have adequate information and knowledge for your age regarding RH, including puberty, pregnancy, and STDs/STIs. | 10.30 | 5.727 | .682 | .815 |
| HAK4-You are well informed about the behavioral risk factors for noncommunicable disease, such as chronic respiratory diseases, cardiovascular diseases, cancer, and diabetes, as well as mental health issues. | 10.28 | 5.781 | .671 | .818 |
| HAK5-You are well informed about the need for health screenings, like breast and pelvic tests for females and blood sugar and cholesterol exams or general checkups, as well as vaccinations. | 10.37 | 5.896 | .668 | .819 |

**Decision making and behavior**

| Reliability Statistics | |
| --- | --- |
| Cronbach's Alpha | N of Items |
| .861 | 7 |

| Item-Total Statistics | | | | |
| --- | --- | --- | --- | --- |
|  | Scale Mean if Item Deleted | Scale Variance if Item Deleted | Corrected Item-Total Correlation | Cronbach's Alpha if Item Deleted |
| DMB1-You can judge when and where you should seek health services. | 16.57 | 10.107 | .611 | .845 |
| DMB2-You can decide what to do and not to do to stay healthy or to protect your health, based on information you get from various sources. | 16.25 | 9.432 | .601 | .846 |
| DMB3-You take care of or prioritize your health every day, based information you have obtained, regardless of the circumstance. | 16.45 | 8.826 | .705 | .830 |
| DMB4-You avoid substances like cigarette, alcohol, and other substances as well as too much sweet diet that are not good for your health. | 16.22 | 9.620 | .653 | .838 |
| DMB5-You protect yourself from unhealthy relationships, unplanned pregnancy, and STDs/STIs. | 16.24 | 9.451 | .619 | .843 |
| DMB6-Whenever you experience any health problem, including RH related problems or STDs/STIs symptoms, you promptly seek help from a health professional. | 16.29 | 9.746 | .595 | .846 |
| DMB7-You accurately follow the health advice, instructions, and directions you receive from a healthcare provider and/or a pharmacist. | 16.23 | 9.416 | .632 | .841 |

**Citizenship and responsibility**

| Reliability Statistics | |
| --- | --- |
| Cronbach's Alpha | N of Items |
| .783 | 3 |

| Item-Total Statistics | | | | |
| --- | --- | --- | --- | --- |
|  | Scale Mean if Item Deleted | Scale Variance if Item Deleted | Corrected Item-Total Correlation | Cronbach's Alpha if Item Deleted |
| CR1-You believe that your health knowledge and understanding are valuable to your family members, friends, and others. | 5.05 | 1.621 | .569 | .766 |
| CR2-You share your health knowledge with friends to help them avoid risky behaviors, such as addiction, and adopt healthy habits. | 5.26 | 1.518 | .655 | .670 |
| CR3-You actively participate in health promotion efforts, such as sanitation activities, health awareness campaigns or clubs, and other initiatives within your school and community. | 5.31 | 1.683 | .648 | .683 |

**S6.3. Test-retest (Intra-class Correlation Coefficients)**

| **Case Processing Summary** | | | |
| --- | --- | --- | --- |
|  | | N | % |
| Cases | Valid | 32 | 100.0 |
|  | Excluded^a^ | 0 | .0 |
|  | Total | 32 | 100.0 |
| a. Listwise deletion based on all variables in the procedure. | | | |

| **Intraclass Correlation Coefficient** | | | | | | | |
| --- | --- | --- | --- | --- | --- | --- | --- |
|  | Intraclass Correlation^b^ | 95% Confidence Interval | | F Test with True Value 0 | | | |
|  |  | Lower Bound | Upper Bound | Value | df1 | df2 | Sig |
| Single Measures | .712^a^ | .611 | .814 | 169.847 | 31 | 2015 | .000 |
| Average Measures | .994 | .990 | .997 | 169.847 | 31 | 2015 | .000 |
| Two-way random effects model where both people effects and measures effects are random. | | | | | | | |
| a. The estimator is the same, whether the interaction effect is present or not. | | | | | | | |
| b. Type A intraclass correlation coefficients using an absolute agreement definition. | | | | | | | |

**Health information competency**

| **Intraclass Correlation Coefficient** | | | | | | | |
| --- | --- | --- | --- | --- | --- | --- | --- |
|  | Intraclass Correlation^b^ | 95% Confidence Interval | | F Test with True Value 0 | | | |
|  |  | Lower Bound | Upper Bound | Value | df1 | df2 | Sig |
| Single Measures | .752^a^ | .656 | .844 | 77.382 | 31 | 713 | .000 |
| Average Measures | .986 | .979 | .992 | 77.382 | 31 | 713 | .000 |
| Two-way random effects model where both people effects and measures effects are random. | | | | | | | |
| a. The estimator is the same, whether the interaction effect is present or not. | | | | | | | |
| b. Type A intraclass correlation coefficients using an absolute agreement definition. | | | | | | | |

**Communication**

| **Intraclass Correlation Coefficient** | | | | | | | |
| --- | --- | --- | --- | --- | --- | --- | --- |
|  | Intraclass Correlation^b^ | 95% Confidence Interval | | F Test with True Value 0 | | | |
|  |  | Lower Bound | Upper Bound | Value | df1 | df2 | Sig |
| Single Measures | .708^a^ | .597 | .816 | 32.538 | 31 | 341 | .000 |
| Average Measures | .967 | .947 | .982 | 32.538 | 31 | 341 | .000 |
| Two-way random effects model where both people effects and measures effects are random. | | | | | | | |
| a. The estimator is the same, whether the interaction effect is present or not. | | | | | | | |
| b. Type A intraclass correlation coefficients using an absolute agreement definition. | | | | | | | |

**Health awareness and knowledge**

| **Intraclass Correlation Coefficient** | | | | | | | |
| --- | --- | --- | --- | --- | --- | --- | --- |
|  | Intraclass Correlation^b^ | 95% Confidence Interval | | F Test with True Value 0 | | | |
|  |  | Lower Bound | Upper Bound | Value | df1 | df2 | Sig |
| Single Measures | .777^a^ | .680 | .865 | 38.855 | 31 | 279 | .000 |
| Average Measures | .972 | .955 | .985 | 38.855 | 31 | 279 | .000 |
| Two-way random effects model where both people effects and measures effects are random. | | | | | | | |
| a. The estimator is the same, whether the interaction effect is present or not. | | | | | | | |
| b. Type A intraclass correlation coefficients using an absolute agreement definition. | | | | | | | |

**Decision making and behavior**

| **Intraclass Correlation Coefficient** | | | | | | | |
| --- | --- | --- | --- | --- | --- | --- | --- |
|  | Intraclass Correlation^b^ | 95% Confidence Interval | | F Test with True Value 0 | | | |
|  |  | Lower Bound | Upper Bound | Value | df1 | df2 | Sig |
| Single Measures | .755^a^ | .657 | .848 | 43.887 | 31 | 403 | .000 |
| Average Measures | .977 | .964 | .987 | 43.887 | 31 | 403 | .000 |
| Two-way random effects model where both people effects and measures effects are random. | | | | | | | |
| a. The estimator is the same, whether the interaction effect is present or not. | | | | | | | |
| b. Type A intraclass correlation coefficients using an absolute agreement definition. | | | | | | | |

**Citizenship and responsibility**

| **Intraclass Correlation Coefficient** | | | | | | | |
| --- | --- | --- | --- | --- | --- | --- | --- |
|  | Intraclass Correlation^b^ | 95% Confidence Interval | | F Test with True Value 0 | | | |
|  |  | Lower Bound | Upper Bound | Value | df1 | df2 | Sig |
| Single Measures | .794^a^ | .694 | .878 | 23.911 | 31 | 155 | .000 |
| Average Measures | .959 | .932 | .977 | 23.911 | 31 | 155 | .000 |
| Two-way random effects model where both people effects and measures effects are random. | | | | | | | |
| a. The estimator is the same, whether the interaction effect is present or not. | | | | | | | |
| b. Type A intraclass correlation coefficients using an absolute agreement definition. | | | | | | | |
